# Understanding the Role of the ‘Self’ in the Social Priming of Mimicry

**DOI:** 10.1371/journal.pone.0060249

**Published:** 2013-04-02

**Authors:** Yin Wang, Antonia F de C Hamilton

**Affiliations:** School of Psychology, University of Nottingham, Nottingham, United Kingdom; Royal Holloway, University of London, United Kingdom

## Abstract

People have a tendency to unconsciously mimic other's actions. This mimicry has been regarded as a prosocial response which increases social affiliation. Previous research on social priming of mimicry demonstrated an assimilative relationship between mimicry and prosociality of the primed construct: prosocial primes elicit stronger mimicry whereas antisocial primes decrease mimicry. The present research extends these findings by showing that assimilative and contrasting prime-to-behavior effect can both happen on mimicry. Specifically, experiment 1 showed a robust contrast priming effect where priming antisocial behaviors induces stronger mimicry than priming prosocial behaviors. In experiment 2, we manipulated the self-relatedness of the pro/antisocial primes and further revealed that prosocial primes increase mimicry only when the social primes are self-related whereas antisocial primes increase mimicry only when the social primes are self-unrelated. In experiment 3, we used a novel cartoon movie paradigm to prime pro/antisocial behaviors and manipulated the perspective-taking when participants were watching these movies. Again, we found that prosocial primes increase mimicry only when participants took a first-person point of view whereas antisocial primes increase mimicry only when participants took a third-person point of view, which replicated the findings in experiment 2. We suggest that these three studies can be best explained by the active-self theory, which claims that the direction of prime-to-behavior effects depends on how primes are processed in relation to the ‘self’.

## Introduction

People have a tendency to unconsciously imitate other's actions, termed “mimicry” [1]. This mimicry plays a critical role in creating social bonds between people and has been regarded as a behavioral strategy for social affiliation [2]–[4]. Although mimicry is not normally conscious controlled, previous studies show that mimicry is a subtle and flexible behavior which is sensitive to multiple social factors such as prosociality, affiliation goals and self-other distinction [5]–[8]. The present paper explores the interaction of these social factors using priming paradigms.

Several studies so far have examined how prosocial or antisocial primes influence mimicry. Increased mimicry following exposure to prosocial or affiliative stimuli has commonly been found, compared to antisocial and non-social stimuli [9]–[13]. For example, by using a test of visual acuity, Lakin and Chartrand [9] exposed participants to subliminal words related to the conception of affiliation (e.g. affiliate/together) and found more mimicry in a subsequent interaction. van Baaren et al., [10] had participants complete a ‘scrambled sentence’ task in which sentences contained affiliative (e.g. group/cooperate) or disaffiliative (e.g. unique/alone) words. They found that more mimicry behavior was induced in affiliative priming conditions than in disaffiliative conditions. Using a novel stimulus-response compatibility approach to measure mimicry, Leighton et al., [11] and Cook & Bird [12], [13] both found that priming sentences contained prosocial words (e.g. sociable/agreeable) increases mimicry while priming sentences contained antisocial words (e.g. rebel/selfish) decreases mimicry.

The dominant explanation of the prosociality priming effects above suggests that prosocial primes directly activate a goal to affiliate [6], [9], [14], [15]. This explanation is based on the goal-activation theory of prime-to-behavior effects [16] which claims that a given prime directly activates a goal, which then automatically leads to pursuit of that goal. For example, when participants are exposed to words related to the concept of affiliation, they activate an affiliation goal and then show more affiliative behavior including more mimicry.

This goal-activation explanation has also been applied to a series of studies where mimicry is increased following a threat to a participant's affiliative needs. For example, people who were primed with unsuccessful affiliation [9], ostracism [17], [18] and social isolation (e.g. feeling too distinct from others, Uldall et al., unpublished data, cited by [5]) mimic a subsequent interaction partner more than people in a control condition. Here, it is claimed that ostracism or isolation primes strongly activate one's goal/desire to affiliate with others, and thus lead to more mimicry behavior. However, if priming either with prosocial concepts (e.g. affiliative words) or antisocial concepts (e.g. disaffiliation threat) can lead to increased mimicry, it becomes hard to make specific predictions about the direction of priming effects.

Looking more broadly at priming of behavior (not just mimicry), an increasing number of studies showed contrast results in prime-to-behavior effects where the prime-induced behavior is the opposite of the primed goal or concept. For example, early studies suggest that priming of the concept of “elderly” caused participants to walk slower and priming of the concept of intelligence caused participants to perform better on an intellectual task [16], [19]. However, Dijksterhuis et al., [20] found that priming with an exemplar of an older person (e.g. the 89 year old Dutch Queen Mother) or an exemplar of an intelligent person (e.g. Einstein) can lead to the opposite effect, with quicker walking speed and worse performance on the intellectual task.

Dijksterhuis et al., [21] suggest these opposing effects can be best understood in terms of how the prime is processed in relation to the self, and Wheeler et al. [22] takes this further in defining the role of the self-concept in the control of prime-to-behavior effects. In their ‘active-self’ model (see a review [23]), it is proposed that the representation of the self-concept has two components, the chronic self-concept and active self-concept. The chronic self-concept concerns all of the self-concept information that is stored in long-term memory, whereas the active self-concept refers to a subset of chronic self-concept content that is currently accessible and active and used to guide behavior. The active self-concept can shift rapidly in response to environmental perceptual inputs, and thus primed constructs could affect behavior by altering the active self-concept.

There are several differences between goal-activation theory [16] and active-self theory [23]. First, the goal-activation theory suggests that prime constructs directly activate goal representations with no intervening processes. In contrast, the active-self theory suggests that the prime-to-behavior effects are mediated by active self-concept. Prime constructs first influence one's understanding of the self, which then activates corresponding behavioral representation. This means that the interplay between the prime and the ‘self’ determines which behavioral representation will guide behavior.

Second, these two theories make different predictions for prime-to-behavior effects. The goal-activation theory predicts that primes should directly activate congruent goals leading to congruent behavior. This theory can only account for cases where behavior is incongruent with the priming material by suggesting that this priming material engaged a different goal. In contrast, the active-self model allows both congruent and incongruent behavior to occur, depending on how the primed construct interacts with the active self-concept. This means that many potential modulators of the active self-concept (e.g. self-comparison, self-relatedness, perspective-taking) can influence that prime-to-behavior effect despite being independent of the primed concept. For example, although priming intelligence-related words such as ‘smart’ usually induces an assimilative self-concept (i.e. ‘I am smart’) and assimilative behavior (i.e. better performance in a following intellectual task), priming concrete and distinct intelligent exemplars such as ‘Einstein’ induces a contrasting self-concept (i.e. ‘I am no Einstein, I am not smart, I am dumb.’) and contrasting behavior (i.e. bad performance in the intellectual task) [20]. Thus, the active-self model permits the prediction of a wide range of effects that are not easily derived from the direct goal-activation framework.

Previous studies examined the active-self model mainly in stereotype-related behavior, e.g. ‘Einstein and intelligent behavior’ [24], ‘elderly exemplars and walking speed behavior’ [25], [26] and ‘African-Americans and aggression behavior’ [27]. In the present paper, we examine if this model is also relevant to priming of stereotype-unrelated behavior such as mimicry. The flexibility of the active-self model suggests that it could provide a powerful means to explain previous mixed results where both prosocial and antisocial primes lead to more mimicry. However, this has not yet been tested directly. Here we report three experiments which examined the effect of prosocial and antisocial primes on mimicry behavior, while maintaining careful control of the self-relatedness of the primes.

Unlike many previous studies of social priming on automatic behaviors where the impact of the prime was measured on a single, natural setting task (e.g. walking speed [19] or number of mimicry actions [9]), our approach in present study builds on the recent finding that mimicry responses can be recorded in more carefully controlled lab tasks (i.e. the stimulus-response compatibility tasks) with multiple trials per participants (see a review paper [28]) and that these lab tasks show the same priming effects as natural encounters [11]–[13], [29]. In particular, we chose the ‘finger-tapping task’ to measure mimicry [30]–[32] where participants had to move their index or middle finger in response to a number while viewing incongruent or congruent finger movements on a computer screen. Previous research found faster responses to congruent than incongruent actions and took this congruency effect as an accurate and reliable measure of mimicry [28]. Here we aim to examine how prosocial and antisocial primes influence this congruency effect, whether via goal-activation or active self-concept.

## Experiment 1

Experiment 1 was primarily a pilot study which aimed to test if priming with pro/antisocial sentences has an impact on mimicry as measured with our finger tapping task. This provides a basic manipulation check of the validity of our approach. Our design is very similar to Leighton et al. [11], though this study was conducted before we were aware of Leighton's findings. Participants first completed a traditional scrambled sentences task (‘the priming stage’) and then took a finger-tapping task (‘the mimicry recording stage’). The scrambled sentences described either prosocial interactions or antisocial interaction between two characters. For example, one prosocial prime was ‘Larry shares his chocolate ice cream with Kitty; one antisocial prime was ‘Eric plays loud music to interrupt Sarah studying’. Non-social, factual sentences were also used as a control condition (e.g. ‘A rainbow is made of seven different colours’). Unlike the between-subjects priming design in Leighton et al. [11], here we used a within-subjects design which presented all priming stimuli (prosocial, antisocial and non-social) to each participant in different blocks, to remove effects due to individual difference in mimicry. We expected that priming with prosocial interactions would give participants the goal to affiliate and lead to stronger mimicry as shown by Leighton et al., [11]. However, our results surprised us and lead us to conduct the studies reported later.

### Participants

Nineteen students (average age 23.8; S.D. 2.81 years; 14 women and 5 men) took part in Experiment 1. All were right-handed, proficient in the English language, had normal or corrected-to-normal vision and naïve as to the purpose of the study. This experiment was approved by the Ethics Committee of the school of psychology of the University of Nottingham. Participants gave written consent to participate in this experiment and were paid for their participation.

### Methods and materials

**Figure 1:**
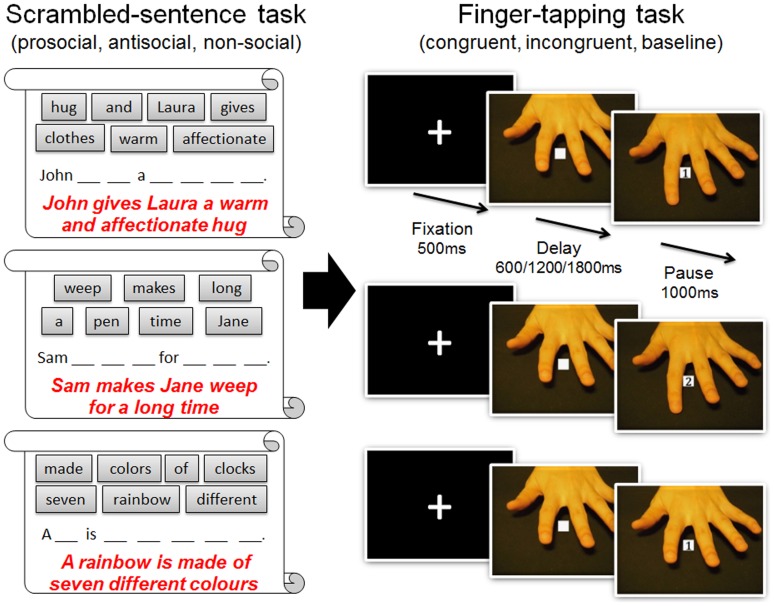
Examples of the priming sentences in the scrambled-sentence task and the hand movement stimuli in the finger-taping task. Each time participants had to complete one page of scrambled sentences describing pro/anti/non-social behaviours on a booklet and then one block of finger tapping task on a computer where they had to respond to a number cue in the middle of the screen and ignored a congruent/incongruent/still hand movement stimuli on the background. They had to complete twelve pages of scrambled-sentence task and twelve blocks of finger tapping task alternately.

The priming manipulation was presented in the form of the “Scrambled Sentence Test” [33] in an A4 booklet. For each test sentence, two words were already presented in the correct order in the answer sheet and participants were required to use the other six words out of a list of seven to construct a grammatically and semantically correct eight-word sentence. Three types of scrambled-sentences were constructed (Figure 1): one was designed to prime prosocial behavior between two fictional characters (e.g., “John gives Laura a warm and affectionate hug”, “Frank and Mary cooperate to make model planes”); another was to prime participants with an antisocial behavior between two characters (e.g., “Sam makes Jane weep for a long time”, “Paul destroys Angelina's new toy train on purpose”), and a third was intended to prime neutral non-social information (e.g., “A rainbow is made of seven different colours”, “London is the capital of the United Kingdom”) (see Text S1 for all sentences).

Participants completed one page at a time from the booklet. Each page contained four sentences and they were all designed to prime the same category of social behaviors (prosocial, antisocial or non-social). Participants could use pencil to write anything on the page for assistance but had to complete the four sentences as quickly as possible. In order to consolidate the priming effect, participants were also required to read their answers to the experimenter for a correction check when they finished each page.

To measure spontaneous mimicry, we used a “finger tapping task” [30], [31], [34]. On each trial, two-frame video sequences of a left hand were displayed on a computer monitor (Figure 1). The first frame showed a left hand resting above a desk, with a white box superimposed on the hand. The second frame showed one of two numbers (1 or 2) on the white box and meanwhile the left hand was performing an finger tapping movement either using index finger or middle finger. The left hand in the video was oriented to appear like a mirror reflection of the participant's own right hand. Using their dominant right hand, participants were instructed to press a key with the index finger if they saw number 1 in the white box, and press a key with the middle finger if they saw number 2 in the white box. They were asked to always respond to the number cue as fast as they can and ignore the hand action in the background. The interval between two frames varied (600, 1200, 1800 ms) to prevent anticipatory responding.

Trials were organized in three types. In congruent trials, the hand in the video frame executed an identical finger movement to the instructed movement (e.g. number 1 + see index finger movement), while in incongruent trials the movement executed by the hand on the screen was different from the instructed movement (e.g. number 2 + see index finger movement). In baseline trials, the hand on the screen did not perform any hand movement, but the number still appeared. Past studies found that observing an action automatically activates the motor representation of that action [31], [32]. Therefore in congruent trials reaction times are facilitated by the mimicry of observed action. Incongruent trials lead to slower responses because the required action must be enforced over the natural tendency to mimic. Mimicry is assessed by calculating the congruency effect (CE)—the reaction time difference between congruent trials and incongruent trials [11]–[13], [28], [35]. There were 12 incongruent trials, 12 congruent trials and 12 baselines in each block of the finger tapping task and they were presented in a pseudo-randomized order.

### Design and procedure

Participants were told that they were taking part in two independent tests of language proficiency and motor control ability, and that the two tests would be alternated to reduce boredom [36]. They had to complete twelve pages of the scrambled-sentence task alternating with twelve blocks of the finger tapping task. The order of the priming pages was fully counterbalanced across participants to prevent practice or carry-over effects impacting on the results. At the end of the study, participants were debriefed to ascertain whether they had guessed the purpose of the experiment.

In order to make participants familiar with these two tasks, they performed a practice session before all the testing sessions. There were three scrambled sentences for practice, each exemplifying one type of priming (prosocial, antisocial and non-social). A short version of the finger tapping task was also prepared for practice, with 5 incongruent trials, 5 congruent trials and 5 baselines trials in a pseudo-randomized order. Cogent running in Matlab was used to present the finger tapping task and record data.

### Results

**Figure 2:**
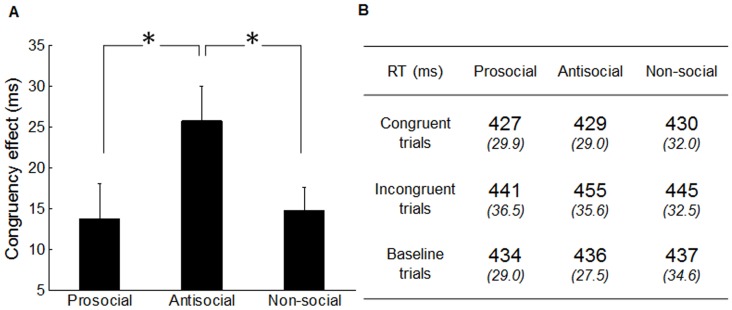
Results in Experiment 1. (A) Mean Congruency Effect (CE) for the three types of priming (prosocial antisocial and non-social). Asterisks represent the statistically significant difference between two bars. Vertical bars indicate standard error. (B) Mean Reaction Time in milliseconds (ms) for participants in each of the three priming groups on congruent, incongruent and baseline trials. Italic numbers indicate standard deviation.

To remove trials where participants did not attend to the number stimuli, incorrect responses (0.10%) were excluded from the analysis, as were all RTs smaller than 100 ms or greater 800 ms (0.48%). To minimize the effect of outliers, we also excluded RTs that were greater than two standard deviations from the conditional means of each participant (0.36%). The congruency effect (CE) for each participant was calculated by subtracting reaction time (RT) in congruent trials from RT in incongruent trials. Figure 2 shows both CE and RT data for each priming group.

In order to test whether mimicry was influenced by the priming sentences in our experimental task, a repeated measures analysis of variance (ANOVA) was conducted on mean RT with congruency (congruent, incongruent, baseline) and primes (prosocial, antisocial, non-social) as variables. The analysis revealed a significant main effect of congruency (F(2,36) = 26.3, p<0.001) with a faster response in congruent trials (*M* = 429 ms, S.E. 30.24) and a slower response in incongruent trials (M = 447 ms, S.E. 35.2); the response in baseline trials was intermediate (M = 436 ms, S.E. 30.5). This main effect of congruency confirmed the success of mimicry measurement in our experimental task. In addition, the ANOVA also revealed a significant interaction between congruency and primes (F(4,72) = 3.52, p<0.011), which suggests that mimicry was modulated by the priming sentences. In order to test whether this congruency × primes interaction was mainly driven by congruent and incongruent conditions, but not the baseline condition, we further conducted a repeated measures ANOVA on baseline trials only, with primes (prosocial, antisocial, non-social) as variables. No significant main effect of primes was found on baseline trials (F(2, 36) = 2.20, p = 0.126), which suggests that the interaction was driven by the congruent and incongruent conditions.

To further examine the priming effect on mimicry, a repeated measures ANOVA was conducted on mean CE with primes (prosocial, antisocial, non-social) as variables. The analysis revealed a significant main effect of primes on CE (F(2,36) = 4.76, p<0.015) (Figure 2A), which is consistent with previous congruency × primes interaction on RT. Specifically, the antisocial priming group induced a stronger CE (M = 25.7 ms, S.E. 18.0) than the non-social (M = 14.8 ms, S.E. 11.8) and prosocial priming group (M = 13.6 ms, S.E. 18.0). Post hoc t-test showed the CE in antisocial priming group is significantly larger than the one in non-social (t (18) = 2.52, p<0.022) and in prosocial priming group (t (18) = 2.81, p<0.012), but there was no difference between the prosocial and non-social priming groups (t (18) = 0.24, p = 0.813).

### Discussion

The results of experiment 1 surprised us. Priming with scrambled sentences describing pro and anti-social behaviors did impact on mimicry, but not in the predicted direction. Our data showed a prime-incongruent effect on mimicry that participants had stronger mimicry following antisocial priming than prosocial and non-social priming (Figure 2A). These results contradict the very similar study by Leighton et al. [11], which found stronger mimicry following prosocial priming in the same task. It is therefore important to understand why our study yielded results which contrast so strongly with Leighton's study.

One possible reason is simply that our study and Leighton's were conducted in different labs with different experimental setups. For example, we used a within-subjects design whereas Leighton used a between-subjects design, and we used finger-tapping task while they used a hand-opening task. These explanations seem unlikely. Instead, we hypothesized that the different results are due to some subtle factors in the priming sentences themselves. Our study only used very concrete prime sentences which were unrelated to the self. Participants read about two people in harmony/conflict but were not engaged in the harmony/conflict themselves (e.g. “John gives Laura a warm and affectionate hug”, “Robin harshly blames the project failure on Amy”). In contrast, Leighton's priming stimuli were more abstract and self-related sentences and participants were to some extent involved in the primes (e.g. “She is my friend”, “We are against this”). The idea that the self-relatedness of the prime influences the direction of the priming effects is not easy to understand under the goal-activation theory, but can potentially be explained by the active-self account. This account emphasizes the importance of the ‘self’ in determining the magnitude and direction of priming effects on behavior [23]. Under this account, the difference in self-relatedness of the priming sentences could lead to assimilative or contrasting prime-to-behavior effects and thus account for the different results between Leighton's study and ours. Experiment 2 tests this possibility.

## Experiment 2

In experiment 2, we aim to examine whether the contrast effects in experiment 1 come from the self-relatedness of the primes. We produced entirely new two sets of scrambled sentences with pro/anti/social primes but manipulated the self-relatedness of those primes. Specifically, the first set described the pro/anti social behaviors from the third-person perspective, just like the sentences in experiment 1 (e.g. “Greg encourages others to be friends with Lauren”, “Joe cruelly bullied Stephanie about her weight problem”). The second set used the same structure, but each sentence was modified by replacing the first character with ‘I’ or ‘we’ and thus presented the behavior from the first-person perspective (e.g. “We encourage others to be friends with Lauren”). In the antisocial sentences, ‘I’ or ‘we’ was always the protagonist rather than the victim of the antisocial behavior (“I cruelly bullied Stephanie about her weight problem”). In this way, all the pro/antisocial behaviors in the first-person and third-person perspective version were identical, except that the former were self-related and the latter were not.

As previous studies found that priming from self-focus or first-person-perspective-taking enhances assimilative behavior whereas priming from other-focus or third-person-perspective-taking enhances contrasting behavior [22], [23], [27], [37]–[40], we predicted that the third-person group would replicate the contrast priming effects in Experiment 1 where more mimicry was induced by third-person antisocial primes than by third-person prosocial primes. In contrast, priming from a first-person perspective should allow the primed concept to be assimilated into the participant's behavior, so that first-person prosocial primes should induce more mimicry behavior. This would replicate the pattern of previous studies where prosocial primes induce more mimicry than antisocial primes [9]–[11].

### Participants

Thirty-two right-handed, native English speaking undergraduate students (average age 20.4; S.D. 1.88 years; 22 women and 10 men) participated in Experiment 2. None of them had participated in Experiment 1. Half of the participants (11 women and 5 men) were randomly assigned to the 3rd person perspective group, the other half to the 1^st^ person perspective group. Again, this experiment was approved by the Ethics Committee of the school of psychology of the University of Nottingham. Participants gave written consent to participate in this experiment and were paid for their participation.

### Methods, materials, design and procedure

These were the same as those in experiment 1, except that two new sets of scrambled-sentence task were prepared for each perspective-taking group. For the third person perspective group, 12 pages of entirely new scrambled sentences were remade: 4 pages of prosocial behavior priming, 4 pages of antisocial behavior priming and 4 pages of non-social priming (see Text S2 for all sentences). For the first person perspective group, we adopted the same sentences but just changed the first character's name into “I” or “We”. The non-social priming sentences in first and third person group were the same.

### Results

**Figure 3:**
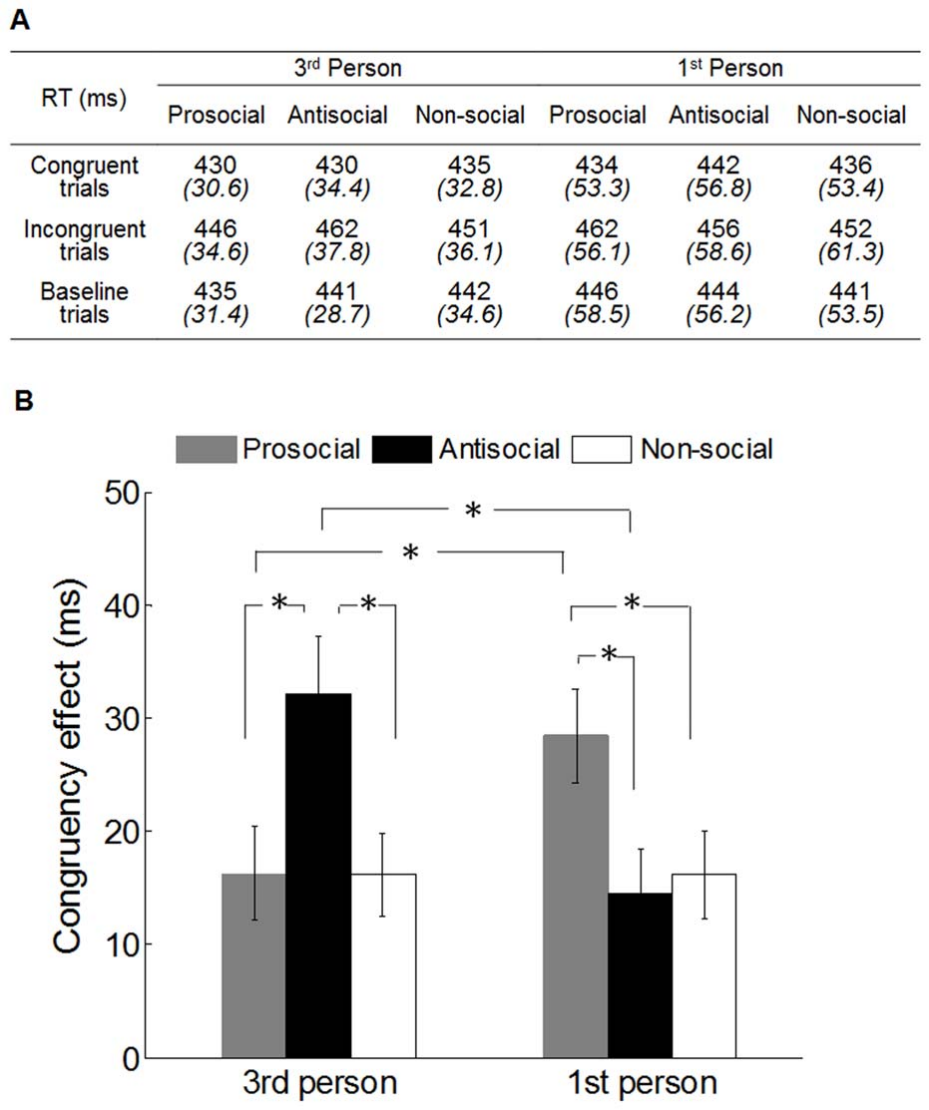
Results in Experiment 2. (A) Mean reaction time in milliseconds (ms) for participants in each of two perspective-taking groups (3rd person and 1st person), each of three priming groups (prosocial, antisocial and non-social), and each of three congruency conditions (congruent, incongruent and baseline trials). Italic numbers indicate standard deviation (B) Mean CE for participants in each of two perspective-taking groups and each of three priming groups. Asterisks represent the statistically significant difference between two bars. Vertical bars indicate standard error.

The same procedure as Experiment 1 was implemented on raw RT data, to remove incorrect responses (0.08%) and RT outliers (0.93%). First, in order to examine whether self-relatedness can alter the priming effects on mimicry, a three-way repeated measures ANOVA was conducted on participants‘ mean RT, with congruency (congruent, incongruent, baseline), primes (prosocial, antisocial, non-social) and self-relatedness (3^rd^-person, 1^st^-person) as variables (Figure 3A). The three-way ANOVA analysis revealed a significant main effect of congruency (F(2,60) = 51.34, p<0.001) and a significant three-way interaction: congruency × primes × self-relatedness (F(4,120) = 4.84, p<0.001). Second, in order to test whether this three-way interaction was mainly driven by congruent and incongruent conditions, but not the baseline condition, we conducted a repeated measures ANOVA on baseline trials only, with primes (prosocial, antisocial, non-social) and self-relatedness (3rd-person, 1st-person) as variables. The result showed no interaction between primes and self-relatedness on baseline trials (F(2,60) = 1.55, p = 0.221), which suggests that the three-way interaction was driven by the congruent and incongruent conditions.

We then performed a two-way ANOVA on participants’ CE with primes (prosocial, antisocial, non-social) and self-relatedness (3rd-person, 1st-person) as variables. In line with the three-way interaction on RT, the two-way ANOVA analysis on CE revealed a significant main effect of primes (F(2,60) = 3.80, p<0.028) and a significant two-way interaction: primes × self-relatedness (F(2,60) = 14.13, p<0.001). These results suggest that the priming effects on mimicry between two perspective-taking groups were significantly different.

In order to further examine the specific priming effect on mimicry in each perspective-taking group, we conducted a repeated measures ANOVA analysis for each group, on mean CE with primes (prosocial, antisocial, non-social) as variables. The analysis revealed a significant main effect of primes on CE in both 3^rd^-person (F(2,30) = 11.87, p<0.001) and 1^st^-person (F(2,30) = 6.59, p<0.004) group (Figure 3B). For 3^rd^-person group, post-hoc t-test showed that the CE in antisocial priming condition was significantly larger than the one in prosocial (t (15) = 5.02, p<0.001) and non-social (t (15) = 3.53, p<0.003) priming condition, which replicated the results in experiment 1. In contrast, post-hoc t-test in 1^st^-person group showed that CE in prosocial priming condition was significantly larger than the one in antisocial priming condition (t (15) = 3.32, p<0.005) and non-social (t (15) = 3.16, p<0.007) priming condition, which was compatible with the findings of Leighton et al. 2010. When we directly compared the priming effects on CE between two self-relatedness groups, we found that antisocial priming effect was significantly larger in the 3^rd^ person group than in the 1^st^ person group (t(30) = 2.87, p<0.007) while prosocial priming effect was significantly smaller in the 3^rd^ person group than in the 1^st^ person group (t(30) = 2.17, p<0.039); the priming effects in two non-social priming conditions were not significantly different (t (30) = 0.002, p = 0.998).

### Discussion

The results in Experiment 2 clearly show that self-relatedness determines the direction of the social priming on mimicry. Just as we predicted, the results in third-person group replicated the contrasting priming effect in experiment 1 with a new group of participants; and the results in first-person group replicated the assimilative pattern in Leighton and previous studies. Specifically, antisocial behavior primes increase mimicry only in the third-person group whereas prosocial behavior primes increase mimicry only in the first–person group. These results demonstrate that the differences between the results of Leighton et al [11] and our experiment 1 are due to the self-relatedness of the primes, and not to other extraneous factors. The data also support the active-self model of priming effects, rather than a goal-activation model. Before considering the theoretical implications of this result in detail, we wanted to confirm its robustness and reliability. In particular, we aimed to test if this same response pattern can be obtained with a different priming method.

## Experiment 3

In Experiment 3, we aimed to test whether the results in Experiment 2 can be replicated by using other priming methods. Instead of using the scrambled sentence task, we developed a novel video priming approach where participants first watched cartoon movies depicting pro-/antisocial behavior between three animate shapes (i.e. helper, hinderer, and bystander) and then completed a story-telling task. This non-verbal priming method is based on the finding that adults and children can perceive the behavior of simple animate shapes in terms of complex interactions such as theory of mind and prosocial behavior [41], [42]. Observation of animate shapes behaving in pro/antisocial fashion can prime pro/antisocial behavior in children [18], [42]. In order to manipulate the self-relatedness of the social primes, here participants were required to take the perspective of one animate shape when watching the video and to describe the story from that shape's point view in the story-telling task. For example, if participants were going to be primed with self-related prosocial behavior, they would be asked to watch a prosocial video from the perspective of the helper and to tell the story from the helper's point of view; in contrast, if participants were going to be primed with self-unrelated antisocial behavior, they would be asked to watch an antisocial video from the perspective of a bystander and to describe the story as they were the bystander shape. Compared to the scrambled sentence task, participants were never exposed to any pre-defined pro/antisocial words; instead, they chose their own way to describe their understanding of the pro/antisocial videos. Therefore, this approach provided a more natural, vivid and ecologically valid way to present social primes.

Unlike the between-subject design of experiment 2 where participants were randomly allocated into two perspective-taking groups, here we used a fully within-subject design for the priming of prosociality and self-relatedness (i.e. each participant had both first and third person perspective when watching the pro/antisocial videos). This allows us to remove possible individual differences in performance, and prepare for future neuroimaging studies. We predict that, like experiment 2, prosocial cartoon primes increase mimicry only when viewed from a first-person perspective and antisocial cartoon primes increase mimicry only when viewed from a third-person perspective.

### Participants

Eighteen right-handed, native English speaking undergraduate students (average age 20.1; S.D. 1.49 years; 11 women and 7 men) participated in Experiment 3. None of them had participated in Experiment 1 or 2. The experiment was approved by the Ethics Committee of the school of psychology of the University of Nottingham. Participants gave written consent to participate in this experiment and were paid for their participation.

### Methods and materials

**Figure 4:**
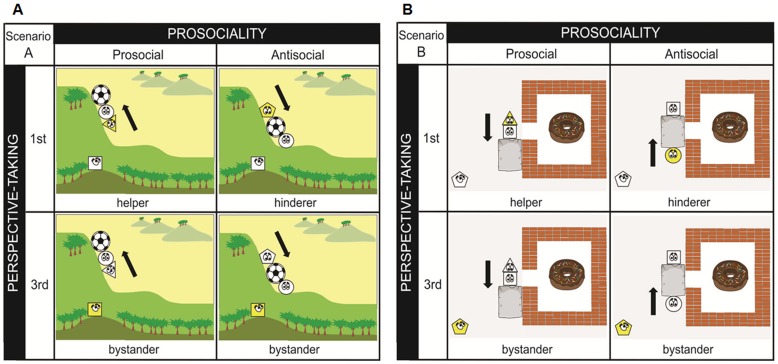
Pro/antisocial cartoons in Experiment 3. (A) In scenario A, participants saw a character (the ‘triangle shape’) initially at the bottom of a hill and attempted to push a football up onto the hill twice, each time falling back to the bottom of the hill. On the third attempt, the ball-pusher was either aided up by a helper (the ‘sphere’ shape) who pushed it from behind (‘prosocial scene’), or was resisted by a hinderer (the ‘pentagon’ shape) who pushed the ball down to the bottom of the hill (‘antisocial’ scene). There was also a bystander (the ‘square’ shape) standing at the top of another hill, watching the whole pro/antisocial behavior happening. (B) In Scenario B, participants saw a character (the ‘square shape’) initially outside of a doughnut house and then repeatedly attempted to get into the house by pushing the stone out of the way to the entrance. On the third attempt, the stone-pusher was either aided by a helper (the ‘triangle’ shape) who pushed it from behind (‘prosocial’ scene), or was resisted by a hinderer (the ‘sphere’ shape) who pushed the stone from the opposite direction (‘antisocial’ scene). There was also a bystander (the ‘pentagon’ shape) standing at the left-bottom corner, watching the whole pro/antisocial behavior happening. In all scenarios, the participant was asked to describe the story from the point of view of the yellow shape, which could be either the helper/hinder (first-person) or the bystander (third-person).

We adopted a cartoon movie paradigm [42] to prime pro/antisocial behavior. Two scenarios of simple social interactions were provided. In Scenario A (see Figure 4A), participants saw a character (the “triangle shape”) initially at the bottom of a hill and then repeatedly attempted to push a football up onto the hill. On the third attempt, the ball-pusher was either aided up by a helper (the ‘sphere’ shape) who pushed it from behind (“prosocial” condition), or was resisted by a hinderer (the “pentagon” shape) who pushed the ball down to the bottom of the hill (“antisocial” condition). In Scenario B (see Figure 4B), participants saw a character (the “square shape”) initially outside of a doughnut house and then repeatedly attempted to get into the house by pushing the stone out of the way to the entrance. On the third attempt, the stone-pusher was either aided by a helper (the “triangle” shape) who pushed it from behind (“prosocial” condition), or was resisted by a hinderer (the “sphere” shape) who pushed the stone from the opposite direction (“antisocial” condition).

There were eight types of cartoon movie (Figure 4) and each movie lasted 30 seconds. Each movie involved three cartoon characters with human-like eyes: a ball/stone pusher, a helper/hinderer, and a bystander (always sits at the left-bottom of the movie). Either the helper/hinderer or the bystander was coloured yellow and the other shapes were white.

### Design and procedure

Participants were ostensibly told that they were going to complete two independent tasks: a story telling task to measure their language ability and a finger tapping task to measure their motor control ability. In the story-telling task, participants were required to imagine themselves as the yellow-colored cartoon character when watching the movie and afterwards to write down the story from that point of view. In half of the movies, the helper/hinderer was yellow-colored, so participants had to write down the pro-/anti-social story from a first-person perspective. In the other half where the bystander was yellow-colored, participants had to write down the pro-/anti- social story from a third-person perspective. To assure the perspective-taking manipulation, participants were asked to describe the story in a pre-defined structure. For example, when participants were watching a prosocial story from a first-person perspective (see Figure 4A, up-left), they had to complete sentences like this: “The white sphere is trying to……; and I am trying to……”; when participants watching an antisocial story from a third person perspective (see Figure 4A, down-right), they had to complete sentences like this: “I am watching……; The white sphere is trying to……; The white triangle is trying to……”. This design allows us to enforce perspective taking without ever using the words “helper” or “hinder” or other pro/antisocial labels to the participants.

Each participant had to complete eight story telling task (2 scenarios×2 pro/antisocial priming×2 perspective-taking) alternating with eight blocks of the finger tapping task. The order of the cartoon movies was fully counterbalanced across participants to prevent practice or carry-over effects impacting on the results. The finger tapping task in Experiment 3 was identical to previous two experiments (i.e. 12 incongruent, 12 congruent and 12 baseline trials in a block of the finger tapping task and they were in a pseudo-randomized order). At the end of the study, participants were debriefed to ascertain whether they had guessed the purpose of the experiment.

### Results and Discussion

**Figure 5:**
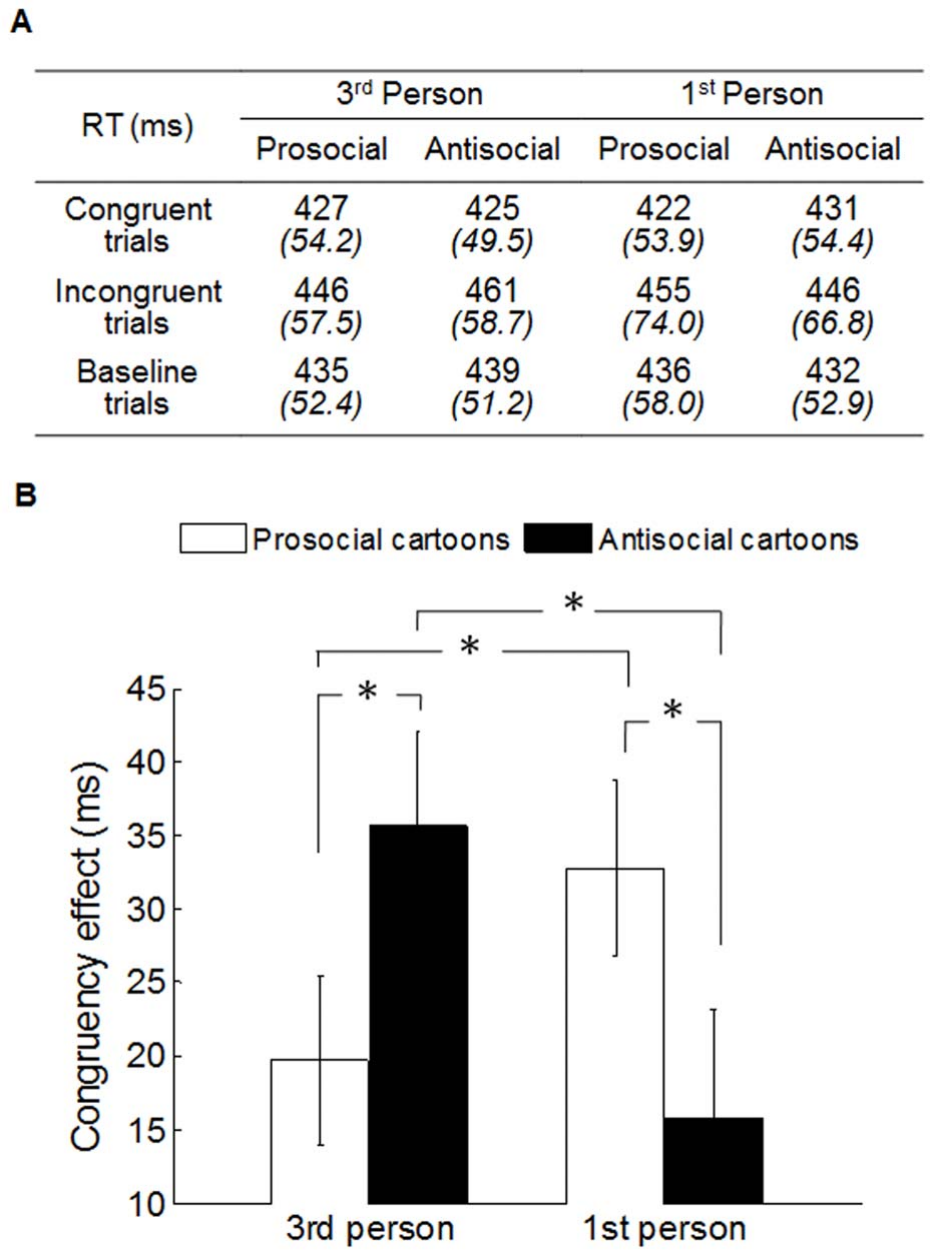
Results in Experiment 3. (A) Mean reaction time in milliseconds (ms) for participants in each of two perspective-taking groups (3rd person and 1st person), each of two priming groups (prosocial and antisocial), and each of three congruency conditions (congruent, incongruent and baseline trials). Italic numbers indicate standard deviation (B) Mean CE for participants in each of two perspective-taking groups and each of two priming groups. Asterisks represent the statistically significant difference between two bars. Vertical bars indicate standard error.

The same procedure as Experiment 1 and 2 was implemented on raw RT data, to remove incorrect responses (0.15%) and RT outliers (0.85%). First, in order to examine whether perspective-taking can alter the priming effects on mimicry, a three-way repeated measures ANOVA was conducted on participants' mean RT, with congruency (congruent, incongruent, baseline), primes (prosocial, antisocial) and perspective-taking (first-person, third-person) as variables (Figure 5A). The three-way ANOVA analysis revealed a significant main effect of congruency (F(2,34) = 19.05, p<0.001) and a significant three-way interaction: congruency × primes × perspective-taking (F(2,34) = 10.17, p<0.001). Second, in order to test whether this three-way interaction was mainly driven by congruent and incongruent conditions, but not the baseline condition, we conducted a repeated measures ANOVA on baseline trials only, with primes (prosocial, antisocial) and self-relatedness (3rd-person, 1st-person) as variables. The result showed no interaction between primes and self-relatedness in baseline condition (F(1,17) = 0.811, p = 0.380), which suggests that the early three-way interaction was driven by the congruent and incongruent conditions.

We then performed a two-way ANOVA on participants' CE with primes (prosocial, antisocial) and perspective-taking (first-person, third-person) as variables. In line with the three-way interaction on RT, the two-way ANOVA analysis revealed a significant two-way interaction on CE: primes × perspective-taking (F(1,17) = 27.59, p<0.001)(Figure 5B). For the third-person group, post-hoc t-test showed that the CE in antisocial condition was significantly larger than the one in prosocial condition (t (17) = 2.42, p<0.027). For the first-person group, the CE in prosocial condition was significantly larger than the one in antisocial condition (t (17) = 3.07, p<0.007). When comparing the pro/antisocial priming effects between two perspective-taking groups, we found that the CE in third-person antisocial condition was significantly larger than the one in first-person antisocial condition (t (17) = 3.89, p<0.001) and the CE in third-person prosocial condition was significantly smaller than the one first-person prosocial condition (t (17) = 2.27, p<0.037). Taken together, these results replicate previous results in Experiment 1 and 2 by using a new priming method, and suggest that the antisocial primes enhance mimicry only in third-person perspective while prosocial primes enhance mimicry only in first-person perspective.

## General Discussion

In the present study, we investigate the underlying mechanism of social priming on mimicry. Three experiments provide converging evidence that the self-relatedness of a prime substantially influences the social priming of mimicry. Specifically, experiment 1 demonstrated a surprising contrast priming effects on mimicry. By using third-person pro/antisocial primes, we found antisocial primes induce stronger mimicry than prosocial primes. In experiment 2, we further verified that priming with first-person prosocial sentences increases mimicry, but priming with third-person antisocial sentences also increases mimicry. Finally, in experiment 3, we replicated the same results of experiment 2 by using a new cartoon priming approach. Again, prosocial primes increase mimicry only when participants take a first-person perspective and antisocial primes increase mimicry only when participants take a third-person perspective. Taken together, these findings provide direct evidence that the self-relatedness of a prime has a critical role in the control of mimicry behavior.

These results are important for several reasons. First they may help us understand why some priming studies yield mixed results, and point to more effective methods for the study of priming. Second, they can help us discriminate between different theories of the control of mimicry and the priming of automatic behavior. Finally, they lead to several new predictions and future directions.

### Methodological Implications

Our findings that prosocial primes do not always enhance mimicry have important methodological implications for future priming research on mimicry. Previous studies only control the prosociality of the scrambled sentences (e.g. contains prosocial or antisocial words), but did not control other factors of the sentences such as first and third person pronoun (e.g. “he”, “they”, “I”, “we”) [10]–[13]. In the current study, our results revealed that self-unrelated primes lead to contrasting effects whereas self-related primes lead to assimilative effects (Figure 3 and 5). This suggests that mimicry is not only sensitive to the pro/antisocial words in the priming sentences, but also the self-relatedness of the primed content. Future studies using tasks like the scrambled sentences task to provide conceptual priming must thus consider the whole meaning of each sentence, not just the presence of key pro/antisocial words. It is possible that failure to control for the self-relatedness of primes could account for at least some of the mixed results and failure-to-replicate in the priming literature [43].

Our paper also validates a non-verbal priming task (i.e. experiment 3, cartoon movie priming) and shows that this task influences behavior in just the same way as a more traditional scrambled sentences task. In this task, participants viewed video clips showing pro/anti-social behavior [42] and then wrote down a description of what they saw from a particular viewpoint. This is potentially useful when studying populations where language ability is more limited, such as children [18] or those with autism [13]. Finally, we show that it is possible to obtain robust priming effects using within-subjects design. Although we found relatively small congruency effects compared to previous studies[11]–[13], these priming effects were reliably replicated across three studies. This opens the way to study the neural mechanisms of social priming using functional MRI.

### Theories of prime-to-behaviour effects

The automatic mimicry of another person's action can be considered a special class of perception-action mapping [44]. Numerous studies over the last decade have examined how different priming contexts have subtle effects on behavior. The dominant explanation of these priming effects is based on the idea of goal-activation [16] which claims that a given prime directly activates a goal, which then automatically leads to pursuit of that goal. Applying this to the case of mimicry and social primes, it is proposed that when participants are exposed to words related to the concept of affiliation, they activate an affiliation goal and then show more affiliative behavior including more mimicry [9], [15].

However, this goal-activation theory cannot easily account for the interaction between the prosocial nature of the prime and the self-relatedness of the prime, which we demonstrate in three studies here. There must be an additional self-related processing step in between the perception of a prime and its impact on behavior. This idea of indirect prime-to-behavior relationship is consistent with the active-self model [23], which proposes that prime-behavior effects are mediated by the concept of the self. This model can account for some prime-incongruent behavior in priming literature where priming with an abstract concept (e.g. “professor”) lead to an assimilative behavior (e.g. higher intelligence performance) while priming with a concrete exemplar (e.g. “Einstein”) leads to a contrasting behavior (e.g. lower intelligence performance) [20].

The active-self theory can also account for assimilative and contrasting priming effects in our data. There are four different conditions which we must consider. First, we suggest that when participants read or imagine a prosocial scenario from a first-person point of view, they assimilative this prosocial attitude into their sense of self and show more mimicry in the subsequent mimicry task. As previous studies using abstract primes always report prime-congruent effects on mimicry (i.e. ‘prosocial prime leads to more mimicry’, [9]–[11], we suggest that this first-person perspective could be the default perspective for abstract stimuli.

Second, we suggest that when participants read or imagine an antisocial scenario from a first-person point of view, the imposed anti-social self conflicts with the participant's default concept of themselves as a prosocial person. This means they reject the feeling of being antisocial and do not change their behavior. Thus, mimicry levels following first-person antisocial priming are similar to non-social priming (Figure 3B). It is worth noting that in Leighton's study, antisocial abstract priming lead to less mimicry than neutral priming, whereas in our study, antisocial first-person priming did not decrease mimicry below neutral priming (see Figure 3B). It is possible that the very concrete first-person antisocial primes used in our study lead to strong conflict with the default prosocial self-concept which causes the primes not to be assimilated [45]. In contrast, when participants are exposed to Leighton's abstract antisocial primes, there is less conflict between their naturally prosocial self and the primed antisocial concept, leading to stronger assimilation of the prime and less mimicry.

Third, we consider the case where participants read or imagine an antisocial scenario from a third-person point of view and then show increased mimicry behavior (experiment 1–3 here). There are two possible explanations. First, exposure to third party conflict might motivate participant to prepare to mend the situation and increase social harmony [46]–[49], and the participant would then show increased mimicry [18]. This is a complex but still primarily goal-motivational account of the results. Alternatively, exposure to third party conflict might lead the participant to engage an implicit self-comparison process (similar to the ‘Einstein’ example) and to feel ‘I am not nasty like that’ [50]. This process would prime participants with a prosocial self-concept (e.g. ‘I would not do that antisocial behavior to others, I am a prosocial guy’) and then lead them showing more mimicry. This is a self-based account of the results. Present data do not entirely distinguish these, but we suggest the active-self account is more parsimonious and more general because it can explain both the present data and previous results [20].

Finally, when participants read or imagine a prosocial scenario from a third-person point of view, they do not need to heal the social situation, nor do they feel the behavior they view is unlike themselves (note that no self-comparison process would be activated here because those prosocial behavior in the primes could be very common in participants' own behavioral repertoire). Therefore, their motivation to mimic and sense of self remain unchanged, and levels of mimicry remain the same as baseline (experiment 1 and 2).

Overall, our data demonstrate that the self-relatedness of a prime is critical in determining how that prime influences behavior. We suggest that the active-self model provides a possible account for this result, and that the influence of primes on behavior cannot be as simple as directly activating a single goal that matches the social valence of the primed concepts. Further study will be needed to determine exactly what additional self-related processes are engaged when primes influence mimicry behavior.

### Future research implications

It is interesting to discuss the relationship between the social priming effects on mimicry and those social/non-social priming effects on executive functions [51], [52]. Although attention is an important factor in the stimulus-response compatibility paradigm (i.e. the finger tapping task), it is very unlikely that the social priming effect on mimicry results from attentional processes (see detailed discussion in [11]). Similarly, although the successful performance of the finger tapping task requires good inhibition in incongruent trials, strong evidence suggests that the effect we found in present studies is different from the priming effects on cognitive control. Brass et al. [53], [54] conducted two studies where they functionally and anatomically dissociate the inhibition of mimicry from cognitive control processes (e.g. Stroop task, go-no go task). Their recent study further suggests that social processes for self-other distinction plays a fundamental role in the inhibition of mimicry [7]. Consistent with that, our findings suggest that the social priming of mimicry is more likely based on specific social processes for the ‘self’ rather than domain-general executive functions.

Our conclusions that social priming of mimicry is mediated by active self-concept are also consistent with recent social priming studies on adolescent and autistic populations. Cook & Bird [12], [13] found adults with autism and young adolescents show less mimicry after prosocial priming than typical adults do. Both adolescents and individuals with autism are considered to have a less mature ‘self’ system which provides self-concept, self-understanding, self-other distinction and self-comparison than typical adults [55], [56]. Thus, it is possible that the reduced of social priming of mimicry in these populations results from weakness of the active-self. Future research need to verify this.

It is also interesting to consider the possible neural mechanism for the social priming effects on mimicry. Past research suggests that medial prefrontal cortex (mPFC) is an important brain region for the social control of mimicry [3]. Social stimuli such as eye gaze modulate mimicry by influencing the neural activity in mPFC [57]. mPFC is also strongly involved in self-related tasks [58]. For example, implicit self-other evaluation and comparison strongly engage mPFC [59], [60]. Moreover, mPFC has been linked to one's prosociality. Activity in mPFC was found to be correlated with daily prosocial behavior [61] and more activities in mPFC predicted more subsequent prosocial behavior [62]. Interestingly, a recent neuroimaging study suggests that mPFC is also the neural substrate for social priming effects on behavior. Bengtsson et al., [36] showed that mPFC is actively engaged when self-esteem primes modulate one's cognitive monitoring ability. Given the fact that mPFC involves in all four processes of social priming, control of mimicry, prosociality and self-relatedness, it appears likely that processing of the prosociality and self-relatedness of a prime takes place in mPFC and the neural activity of mPFC determines the pro/antisocial priming effects on mimicry. Future research could investigate this.

### Limitations

There are several limitations in the present studies that need future research to further investigate. First of all, we only primed the participant to be the victim, rather than the protagonist in the first-person antisocial prime. It would be interesting to examine how people mimic when they are primed to be the victims of an antisocial event. Second, across three studies, pro/antisocial primes always increased mimicry relative to non-social primes, and did not ever decrease mimicry below that elicited by non-social primes. It could be interesting to investigate what (if any) social primes can make participants mimic less than non-social situations. Recent studies suggest social group membership as a strong modulator of mimicry where priming social in-groups increases mimicry while priming social out-groups decreases mimicry [63]. As we did not distinguish the ethnicity of our participants in three studies, it will be interesting to see whether Caucasian/African-originated participants have different mimicry patterns or whether the hand stimuli when changed into a black colored skin would elicit different mimicry responses.

### Conclusions

Overall, our series of three studies demonstrate that priming can influence mimicry responses in controlled lab situations, but that the direction of the effects obtained depends critically on the self-relatedness of the primes. We suggest these results are compatible with an active-self model of prime-to-behaviour effects, and that further study of the role of the self in priming would be valuable.

## Supporting Information

Text S1 Scrambled sentences in Experiment 1.
(DOC)

Text S2 Scrambled sentences in Experiment 2.
(DOC)
