# Supplementary material for: Understanding the Role of the ‘Self’ in the Social Priming of Mimicry
Source: PLoS One. 2013 Apr 2;8(4):e60249. doi: 10.1371/journal.pone.0060249 (PMC3614954; doi:10.1371/journal.pone.0060249)
Supplement: Text S1 — Scrambled sentences in Experiment 1. (DOC) [file pone.0060249.s001.doc]

**Scrambled sentences in Experiment 1**

*Pro-social*

1. Paul helps Ivy to fix her old bicycle
2. Mark looks after Josie when she is ill
3. Andrew and Wendy collaborate on their schoolwork happily
4. Matthew gives Jill a flower as a present
5. John gives Laura a warm and affectionate hug
6. Frank and Mary cooperate to make model planes
7. Derek and Nancy have a nice long conversation
8. Jackson and Lisa go cycling every Saturday afternoon
9. Neil helps Jane paint her living room blue
10. Larry shares his chocolate ice cream with Kitty
11. Tina and Arthur bake cakes for their friends
12. Lee invites Kelly to dance at the party
13. Jason and Lauren celebrate their double tennis victory
14. Jerry and Linda play card games all day
15. Alex and Zoe enjoy their holiday in Hawaii
16. Colin and Rebecca clean their messy room together

*Anti-social*

1. Stuart and Eva fight over the last biscuit
2. Richard complains about Alice’s endless whining and moaning
3. Bowen tells Isabel she is boring and stupid
4. Steven and Estelle often race against each other
5. Paul destroys Angelina’s new toy train on purpose
6. Billy feels bullied and insulted by Jane’s sarcasm
7. Sam makes Jane weep for a long time
8. Tom snatches the strawberry lollipop from Mary’s hand
9. Jane and William dispute the money they earn
10. Robin harshly blames the project failure on Amy
11. Kevin scribbles on Betty’s favourite picture book deliberately
12. Nick punches Kate in public without any reasons
13. Tom and Diana quarrel endlessly over their chores
14. Eric plays loud music to interrupt Sarah studying
15. Carry and Jack rival for the Gold Medal
16. Tommy and Ellie work to outwit each other

*Non-social*

1. London is the capital of the United Kingdom
2. Bats are the only mammals which can fly
3. China has the most population in the world
4. A rainbow is made of seven different colours
5. Spring is the first season of the year
6. Michael Jordan is the greatest basketball player in history
7. London will hold the Olympic Games in 2012
8. Fish and chips are a traditional British food
9. Photoshop is a software used to process images
10. The first airplane was invented by Wright Brothers
11. Some birds cannot fly because of their weight
12. The world has five continents and four oceans
13. Kiwi fruit is a natural source of Vitamin C
14. The Nobel prize ceremony is held in Sweden
15. Boots is a high street pharmacy from Nottingham
16. A hearing aid is designed to amplify sounds
