# Supplementary material for: Understanding the Role of the ‘Self’ in the Social Priming of Mimicry
Source: PLoS One. 2013 Apr 2;8(4):e60249. doi: 10.1371/journal.pone.0060249 (PMC3614954; doi:10.1371/journal.pone.0060249)
Supplement: Text S2 — Scrambled sentences in Experiment 2. (DOC) [file pone.0060249.s002.doc]

**Scrambled sentences in Experiment 2** (Here we only show the third person version. In the first person version, the first name of each sentence was replaced by the pronoun ‘I’)

*Pro-social:*

1. Patrick shares sweets with Lola and her friends
2. Harrison helps Julie to understand difficult maths problems
3. Neil and Danielle always play games together happily
4. Ben and Annie motivate each other when jogging
5. Steve and Zoe complete their maths project together
6. Penny enjoyed a long holiday abroad with Callum
7. Chloe planned a surprise birthday party for Owen
8. Melanie comforts Brian with hot chocolate and biscuits
9. Rose and Peter work together to achieve success
10. Sam and Joe play football all afternoon together
11. Mark congratulates Jessica on her wonderful dance performance
12. Dan trusts Theresa to keep his deepest secrets
13. Emma helps Dorothy with the gardening every week
14. Sally and Warren volunteer with children in hospitals
15. Carlos babysits every Friday for Grace’s little sister
16. Greg encourages others to be friends with Lauren

*Anti-social:*

1. Alex and Jane fight over the television channel
2. Dave stole Rachel’s wallet when she wasn’t looking
3. Gareth made Mary cry repeatedly all night long
4. Eddy insulted Susan’s taste in classical Russian opera
5. Tom always refused to eat Alice’s home-made cake
6. Joe cruelly bullied Stephanie about her weight problem
7. Emma slapped Mike when he cheated on her
8. Jenny was annoyed at Ryan for being late
9. Laura was angry at Jordan for being mean
10. Pippa challenged Jack to a fight after school
11. Holly made Josh feel unhappy about his job
12. Nina and Adam shouted loudly at each other
13. Ian scribbles on Betty’s favourite picture book deliberately
14. Aiden and Rebecca disagree over choosing a film
15. Zoe tells Tillie she is lazy and stupid
16. Tess refuses to help Zack with his project

*Non-social:*

1. Yorkshire pudding and horseradish go with roast beef
2. H2O is the chemical property name for water
3. There are 366 days in each leap year
4. Washington is the capital of the United States
5. Humans are distant relatives of apes and chimps
6. Snow White was the first Disney feature animation
7. Mercury is the closest planet to the Sun
8. The light bulb was invented by Thomas Edison
9. Barack Obama is the USA’s first black president
10. Valentine’s day falls on the fourteenth of February
11. A sea breeze is strongest at about 3pm
12. Today the UK population is over 60 million
13. Mandarin is the most commonly spoken world language
14. Romeo and Juliet was written by William Shakespeare
15. The Queen’s Golden Jubilee took place in 2002
16. China won 100 medals at the Beijing Olympics
